# Prenatal Diagnosis of Cardiac Diverticulum with Pericardial Effusion in the First Trimester of Pregnancy with Resolution after Early Pericardiocentesis

**DOI:** 10.1155/2015/154690

**Published:** 2015-10-08

**Authors:** Raquel Garcia Rodriguez, Azahara Rodriguez Guedes, Raquel Garcia Delgado, Lourdes Roldan Gutierrez, Margarita Medina Castellano, Jose Angel Garcia Hernandez

**Affiliations:** Prenatal Diagnosis and Fetal Therapy Unit, Department of Obstetrics and Gynecology, University Maternity Hospital of Canaries, Las Palmas, 35004 Gran Canaria, Spain

## Abstract

Cardiac diverticulum is a rare anomaly, which may present in association with pericardial effusion. Only few cases diagnosed during fetal life have been published and only in 12 cases pericardiocentesis was made with good postnatal outcomes in 83% of the cases. In the first trimester of pregnancy only 6 cases were reported. We described the largest series of cases published. We describe a case of cardiac diverticulum complicated with pericardial effusion during the first trimester of pregnancy and resolved by intrauterine pericardiocentesis at 17 weeks of pregnancy. We made a systematic review of the literature with the cases reported of cardiac diverticulum, management, and outcomes.

## 1. Introduction

Cardiac diverticulum is a rare anomaly, which may present in association with pericardial effusion and may produce complications such as pulmonary hypoplasia or hydrops fetalis. Only a few cases diagnosed during fetal life have been published and 10 cases during the first trimester of pregnancy.

We describe a case of cardiac diverticulum complicated with pericardial effusion, which was diagnosed at 14 weeks of pregnancy and resolved by intrauterine pericardiocentesis at 17 weeks of pregnancy.

We review the ultrasound findings, management, and outcomes in 34 cases published in the literature, 21 of which were associated with pericardial effusion, and in 12 cases a pericardiocentesis was performed. In this group of pericardiocentesis, 10 cases have good postnatal outcomes.

In light of the good neonatal outcome of this procedure, we propose that early pericardiocentesis should be considered as a therapeutic option in these cases that do not resolve spontaneously.

## 2. Case Report

**Figure 1:**
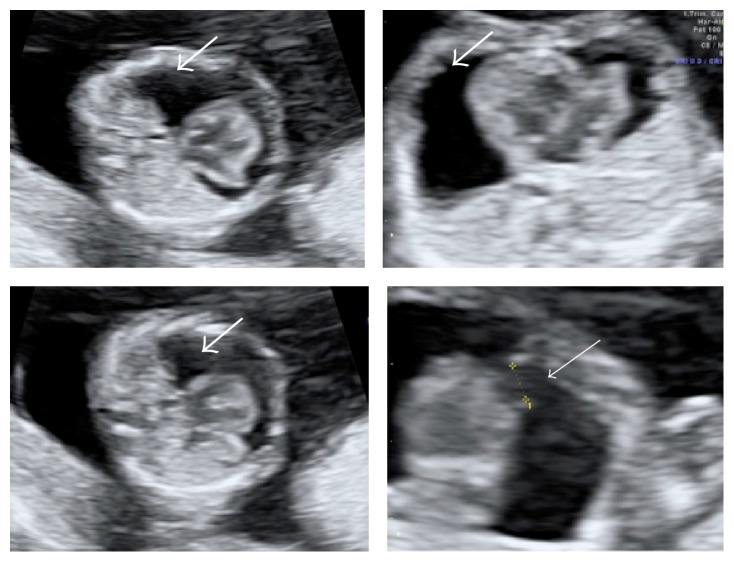
Transversal view of the thorax during the 14th week of pregnancy, where severe pericardial effusion (thick arrow) and cardiac diverticulum in the ventricular apex (thin arrow) can be observed.

A 22-year-old sub-Saharan woman, gravida 2 para 1, was referred to the Unit of Prenatal Diagnosis and Fetal Therapy at 12 + 4 weeks of gestation for a fetal hydrothorax. The ultrasonographic study revealed the presence of severe pericardial effusion. The first trimester screening showed 2.9 mm nuchal translucency, reversed ductus venosus a-wave, absent nasal bone, and absence of tricuspid regurgitation. All other anatomical and echocardiographic findings were normal. Biochemical markers were *β*-hCG 0.14 MoM and PAPP-A 0.72 MoM. The screening results indicated high risk of chromosomopathy. Therefore, a chorionic villus samplingbiopsy study was performed, which revealed a normal 46 XY karyotype. On week 14 of pregnancy, pericardial effusion was still observable. Furthermore, a two-millimeter anechoic image was found on the heart apex, with a narrow base and expanding from the ventricular wall into the pericardium. Blood flow could be observed through this structure with color Doppler sonography. On this basis, the diagnosis of a cardiac diverticulum was made (Figure 1). The rest of the echocardiography was normal.

**Figure 2:**
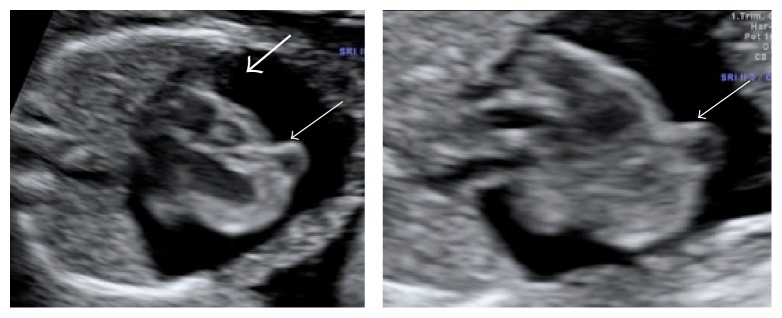
Transversal view of the thorax during the 16th week of pregnancy. Severe pericardial effusion (thick arrow) and cardiac diverticulum in the ventricular apex that connects with the ventricle (thin arrow) can be observed.

Serial ultrasound studies were conducted until week 17 of pregnancy (Figure 2). Given that the pericardial effusion persisted, pericardiocentesis with a 20-gauge needle (like amniocentesis) was performed and a clear serum-like fluid was removed. After pericardiocentesis, both lungs expanded and only a small amount of residual fluid remained. Results of the infections study of the amniotic fluid (*Toxoplasma*, cytomegalovirus, rubella, herpes virus, and parvovirus B19) were negative. A week after pericardiocentesis, no new pericardial effusion was detected. The clinical picture remained stable until week 25, when cardiomegaly and thickening of the ventricular walls were observed, thus leading to a diagnosis of hypertrophic myocardiopathy. Ultrasound studies showed normal ventricular function without signs of cardiac failure. On week 40, a boy was born in eutocic delivery, with 3150-gram body weight, 9/10 Apgar score, and 7.29 umbilical artery pH. The newborn was admitted to hospital for cardiological study. Subsequent serial examinations ruled out hypertrophic myocardiopathy, although they showed residual pericardial effusion, an apical cardiac diverticulum, persistent small ductus venosus, and permeable oval foramen. At present, the child is four years old, remains asymptomatic, and is on a prophylactic treatment with acetylsalicylic acid. He is being monitored in annual follow-up visits to the Service of Pediatric Cardiology.

## 3. Discussion

A cardiac diverticulum is a protrusion located on the ventricular wall, in close communication with it. This rare entity has been scarcely reported in the literature. Prenatal diagnosis may be difficult when it appears isolated [1]. Since such formations are closely communicated with the heart ventriculum, bidirectional blood flow may be observed through them with color or pulsed Doppler sonography [1].

Two types of diverticula are known: apical and nonapical [2, 3]; their characteristics are shown in Table 1. Apical diverticulum of the left ventriculum may be of three different types: apical isolated diverticula, which are not associated with other malformations; multiple diverticula, which are located on the diaphragmatic or anterior surface of the ventriculum; and large apical diverticula, which are associated with midline thoracoabdominal malformations or with the Pentalogy of Cantrell [4]. Although their etiology is not known, they seem to be caused by local weakening of the ventricular wall, which may in turn be due to possible embryogenesis defects [5], secondary to infections, or caused by local ischemia resulting from coronary anomalies, such as stenosis, hypoplasia, intimal proliferation, and thrombosis [6–8]. Differential diagnosis should include aneurisms (Table 1) [6, 9–12], myocardiopathy, Ebstein's anomaly, and auriculoventricular regurgitation [9].

We made a systematic review of the literature with PubMed and Embase database search in English, French, or German which was performed without any restriction of publication date or journal, using the following key words: fetal cardiac diverticulum. The last search was updated in December 2012.

For each included case we focused on the following criteria: time of diagnosis, pericardial effusion association, management, intrauterine evolution, associated complications, and time of delivery fetal outcome (intrauterine death/stillbirth, death after delivery, and survival). We excluded the cases of cardiac aneurysm. After reviewing 27 articles, 34 cases published between 1990 and 2012 met the above criteria in which cases of fetal cardiac diverticulum were presented (Table 2) with our case included.

It was most frequently diagnosed during the second trimester of pregnancy (51%). During the first trimester, 29% of cases were diagnosed; three of them presented increased nuchal translucency. This entity affects male fetuses more than female ones (3 : 1); its most frequent location is on the right ventriculum (70%) and the most frequently involved area is the heart apex (57%). Ultrasonographic findings associated with diverticula include pericardial effusion, cardiomegaly, septal defects and arrhythmia with fetal death before delivery, and hydrops [6, 13, 14]. Pericardial effusion is the most frequently associated finding (63%) and should be considered an indirect sign of the presence of cardiac diverticula. Although the etiology of effusion is not known, it has been proposed to result from the diverticulum rubbing the pericardial walls or from heart failure. Thus, the observation of pericardial effusion makes it necessary to examine the cardiac function [1, 6, 15].

A problem associated with pericardial effusion is that the resulting compression may produce heart failure and pulmonary hypoplasia. The management of such cases (Table 3) usually varies from performing pericardiocentesis to adopting an expectant approach. In a published series of 22 cases, pericardiocentesis was performed in 12 (56%) fetuses of 14 to 25 weeks' gestational age, with an outcome of 10 cases that progressed favorably (83%) and 2 intrauterine deaths (17%) [16, 17]. In the cases of fetal death, the pericardial fluid was blood. In the same series, 7 cases were managed with an expectant approach (32%). Five of them showed spontaneous resolution (71%) and 2 resulted in intrauterine death (29%): one of them, which occurred on week 26, was associated with trisomy 18 and the other, which occurred on week 29, was associated with treated twin-to-twin transfusion syndrome and death of one of the twins after treatment [6, 18].

Our review is the largest series published in the literature with 34 cases of fetal cardiac diverticula. If we exclude the cases of termination of pregnancy, chromosomal abnormalities, and pregnancy complications (twin-to-twin transfusion syndrome), the cardiac diverticulum is a benign condition with good postnatal outcomes, with a mortality of 0.6%. In the cases with pericardial effusion, a pericardiocentesis, performed from 16 weeks of gestation with a 20-gauge needle, seems to be a safe procedure with good outcomes in 83% of cases. The evolution of the pericardial effusion and the diverticula (spontaneous resolution, stability, or progression) is not known because there are few cases published, but the risk of cardiac insufficiency, hydrops fetalis, or pulmonary hypoplasia could be avoided with this procedure.

In our case we made an early diagnosis and treatment with pericardiocentesis with resolution of the pericardial effusion with an excellent outcome. Only 10 cases (29%) were reported during the 11–14 weeks' scan and only 6 cases (17%) with pericardial effusion associated during this period.

Cardiac diverticula are rarely associated with chromosomopathies especially when other malformations are not present [5]. Cardiac abnormalities usually associated with diverticula include ventricular or auricular septal defects, permeable oval foramen, tricuspid atresia, tetralogy of Fallot, persistent left superior vena cava, pulmonary artery hypoplasia, and coarctation of the aorta [5]; cardiomegaly is the most frequently associated complication in the third trimester, like in our case [6, 19]. Usually associated extracardiac malformations include midline thoracoabdominal defects (omphalocele, ectopia cordis, Pentalogy of Cantrell, etc.) [2, 5, 8, 20–22].

The prognosis of this entity is generally good, although the outcome largely depends on the size and location of associated anomalies. Cases of rupture, both pre- and postnatal, arrhythmia, fetal death, heart failure, and coronary insufficiency have been described [9, 16, 18, 21, 23]. In these patients, serial control examinations are necessary to detect possible complications. In general, postnatal progression is good and surgery is not necessary in asymptomatic cases [19].

In conclusion, a cardiac diverticulum is a rare entity. The most frequently associated complication is pericardial effusion, which may result in cardiac decompensation, hydrops fetalis, or pulmonary hypoplasia. Although spontaneous resolution of effusion has been reported, pericardiocentesis is a safe and effective technique, which may be used to reduce the risk of secondary disorders in selected cases. Nowadays there are few cases published and the evidence is poor to establish the appropriated management.

## Figures and Tables

**Table 1:** Differential diagnosis of the different types of diverticulum and aneurism.

	Isolated apical diverticulum	Nonapical diverticulum	Aneurism
Etiopathogeny	Embryogenesis defect	Congenital or acquired focal defect in the muscle wall (viral infection, injury to coronary artery, etc.)	Congenital or acquired focal defect in the muscle wall (viral infection, injury to coronary artery, etc.)

Implantation base on ventriculum	Narrow base	Narrow base	Wide base

Size	Small	Small	Large

Development during pregnancy	Constant size throughout pregnancy	Constant size throughout pregnancy	Enlarging with gestational age

Histology	Myocardium in walls; usually presenting the three layers (myocardium, pericardium, and endocardium)	Myocardium in walls, usually presenting the three layers (myocardium, pericardium, and endocardium)	Myocardium disruption, usually presenting thin myocardium and fibrous tissue

Kinesis	Normal	Normal	Akinetic, hypokinetic, and dyskinetic

Contractility	Contractility in synchrony with heart's rhythm	Contractility in synchrony with heart's rhythm	Contractility paradoxical with heart's rhythm

Complications	Depending on the associated anomalies	Usually not occurring	Arrhythmia, thromboembolism, heart failure, and rupture

Prognosis	Good	Good	Bad

**Table 2:** Description of the cases of cardiac diverticulum reported in the literature.

	Author	GA di	Size	Sex	Location	Karyotype	Associated anomalies	Intervention	Prenatal progression	Neonatal	Follow-up
1	Kitchiner et al. (1990) [13]	33	—	Female	Apex VI	—	Cardiomegaly	No	Stable	Vaginal delivery 40 w; cardiomegaly, tachypnea, heart murmur, muscular IVC, and mild mitral regurgitation	Asymptomatic at 3.5 months of life

2	Hornberger et al. (1994) [9]	31	—	—	Lateral wall below tricuspid valve (RV)	—	—	No	Stable	Vaginal delivery at term	Asymptomatic at 12 months of life

3	Carles et al. (1995) [24]	13	—	Male	Apex LV	—	Pericardial effusion	TOP 14 w	—	—	—

4	Cesko et al. (1998) [25]	17	—	Male	Apex RV	46XY	Pericardial effusion	TOP 22 w	Stable	—	—

5	Cavallé-Garrido et al. (1997) [6]	20	Large	Female	Lateral wall below mitral valve (LV)	Trisomy 18	Ventricular septal defect, hydrops	No	Fetal death 26 w	—	—

6	Cavallé-Garrido et al. (1997) [6]	19	Small	Female	Apex RV	—	No	No	Stable spontaneous resolution at 34 w	Asymptomatic	Asymptomatic at 22 months of life

7	Cavallé-Garrido et al. (1997) [6]	19	Small	—	Apex RV	—	Pericardial effusion	PC 20 w	Stable	Asymptomatic	Asymptomatic at 12 months of life

8	Cavallé-Garrido et al. (1997) [6]	36	Small	Male	Lateral wall below tricuspid valve (RV)	—	Pericardial effusion				Asymptomatic at 18 months of life

9	Johnson et al. (1996) [16]	19	3 mm	Female	Apex RV	46XX	Pericardial effusion	PC 20 w	No relapse after PC, no growth	Eutocic delivery 41 w; weight 3700 grams; asymptomatic	Asymptomatic at 16 months of life

10	Brachlow et al. (2006) [23]	32	—	—	Apex LV	—	Cardiomegaly	No	Stable	—	Asymptomatic at 6 months of life

11	Bernasconi et al. (2004) [26]	22	10 × 5 mm	Male	LV lateral wall below mitral valve^*∗*^	46XY	Pericardial effusion	PC 22 w	—	Fetal death 26 w, probably due to diverticulum rupture	—

12	McAuliffe et al. (2004) [27]	13	4 × 6 mm	Male	Apex RV	46XY	First trimester NT 4.2 mm Pericardial effusion	PC 16 w	Resolution of the effusion; CD stable	Eutocic delivery 38 w; weight 3070 grams; asymptomatic	Asymptomatic at 10 months of life

13	McAuliffe et al. (2004) [27]	13	4 × 3 mm	Male	Apex RV	46XY	First trimester NT 2 mm Pericardial effusion	PC 14 w	Resolution of the effusion; CD stable	Eutocic delivery 38 w; weight 3150 grams; asymptomatic	Asymptomatic at 8 months of life

14	Prefumo et al. (2005) [1]	14	5 × 5	Male	Apex RV	46XY	First trimester NT 3.7 mm; pericardial effusion, ascites, and skin edema	PC 16 w	Resolution of the effusion and hydrops; CD stable; mild cardiomegaly	Vaginal full-term eutocic delivery; asymptomatic	Asymptomatic at 22 months of life

15	Prefumo et al. (2005) [1]	12	1 mm	—	Apex RV	—	First trimester NT 1.2 mm Pericardial effusion	No	Spontaneous resolution of PE with 21 w; CD stable	Full-term eutocic delivery, asymptomatic	Asymptomatic at 17 months of life

16	Gardiner et al. (2005) [19]	14	2-3 mm	—	Apex RV	Normal	Pericardial effusion	PC 14 w	Resolution of the effusion and hydrops; CD collapsed	Asymptomatic at birth	—

17	Gardiner et al. (2005) [19]	14	2-3 mm	—	Apex RV	Normal	Pericardial effusion	TOP	—	—	—

18	Del Río et al. (2005) [18]	13	5 × 5	Female	Apex RV	46XX	Pericardial effusion, septal defect AV^*∗∗*^	No	Spontaneous resolution at 28 w	Eutocic delivery 40 w; weight 3400 grams, asymptomatic at birth	Correction of septal defect at 3 months of life, resection of diverticulum; asymptomatic at 8 months of life

19	Wax et al. (2007) [14]	20	6 × 9 mm	Male	Junction base RV-infundibulum	—	No	No	Stable	Full-term eutocic delivery; weight 3689 grams; asymptomatic; small permeable FO	Asymptomatic at 18 months of life

20	Koshiishi et al. (2007) [17]	24	7 × 10 mm	—	Lateral wall below tricuspid valve (RV)	—	Mild pericardial effusion; MC pregnancy with laser intervention for TTTS at week 20 where donor fetus died	No	Stable	Prenatal fetal death at 29 w	—

21	Pradhan et al. (2007) [28]	28	—	—	Apex LV	—	Fetal arrhythmia Hydrops fetalis	Medical treatment (digoxin)	—	Vaginal delivery 40 w	Asymptomatic at 12 months of life

22	Barberato et al. (2009) [29]	16	5 × 5.7 mm	—	Apex LV	—	Mild pericardial effusion	PC 20 w	Discrete enlargement of PE with normal heart function	Prenatal fetal death 37 w	—

23	Barberato et al. (2009) [29]	30	12 × 13 mm	—	Mitral subvalvular	—	LV dilatation and reduced systolic function	No	Stable	—	Asymptomatic at 6 months of life

24	Davidson et al. (2006) [15]	20	—	—	Apex RV	—	Pericardial effusion	No	Spontaneous resolution	—	Surgical treatment

25	Williams et al.(2009) [3]	21	5 × 5.5 mm	Male	RV	—	Pericardial effusion	PC 24 w	Mild tricuspid regurgitation at 31; CD stable	Full-term delivery	Asymptomatic at a year of life

26	Perlitz et al. (2009) [30]	22	7 × 4 mm	Male	RV lateral wall	—	No	No	Stable, CD growth up to 9 × 9 mm	Eutocic delivery week 40; weight 4010 grams; asymptomatic at birth	Asymptomatic at a year of life

27	Menahem (2010) [31]	19	—	—	Apex LV	—	Pericardial effusion	—	No controls performed	Full-term live birth	Asymptomatic at 10 months of life

28	Carrard et al. (2010) [32]	13	2.6 × 2.9 mm	Male	RV lateral wall	46XY	First trimester NT 2.2 mm Pericardial effusion	PC 17 w	Resolution after PC; CD collapsed at 26 w	Eutocic delivery 40 w, 2780 grams	Asymptomatic at 11 months of life

29	Abi-Nader et al. (2009) [2]	22	3-4 mm	Male	RV	46XY	Pericardial effusion	No	Resolution at 32-33 w	PROM 34 w; Intubation due to prematurity; caesarean section; weight 2460 gr; 2 muscle IVCs	Asymptomatic at 14 months of life

30	Abi-Nader et al. (2009) [2]	21	11 × 15 mm	Male	RV lateral wall below tricuspid valve	—	Isolated	—	—	Eutocic delivery; weight 2780 gr; asymptomatic at birth	Asymptomatic at 16 months of life

31	Abi-Nader et al. (2009) [2]	25	26 × 16 mm (37 s)	Male	RV	—	Arrhythmia and reduced systolic function	Induced delivery	—	Caesarean section 38 + 5 w; weight 3270 grams; mild reduction of systolic function and premature ventricular contractions at birth	Asymptomatic at 3 years of life, on prophylactic treatment with acetyl salicylic acid

32	Williams et al. (2009) [3]	17		—	Apex LV	Normal	Mesocardia, perimembranous IVC	No	Stable	Full-term live birth	Asymptomatic at 2 years of life

33	Paoletti and Robertson (2012) [20]	21	1.6 × 0.4 mm	—	Apex LV	Normal	Defect on thoracoabdominal midline	TOP	—	—	—

34	Nam et al. (2010) [21]	31	12 mm (postnatal)		RV lateral wall below tricuspid valve	—	—	No	Ventricular septal defect	Full-term live birth; asymptomatic at birth; symptoms at 45 days of life: closure of septal defect at 3 months of life	Asymptomatic at 10 months of life

35	Our case	14	2 mm	Male	Apex RV	46XY	Pericardial effusion	PC 17 w	PE resolution after treatment; CD stable; moderate cardiomegaly; normal heart function	Full-term live birth; spontaneous eutocic delivery 40 + 1 w; weight 3150 grams	Asymptomatic at 4 years of life

GA di: gestational age at diagnosis; RV: right ventriculum, LV: left ventriculum; w: weeks of pregnancy; TOP: termination of pregnancy; PC: pericardiocentesis; CD: cardiac diverticulum; IVC: interventricular communication PE: pericardial effusion; PROM: premature rupture of membranes; NT: nuchal translucency.

^*∗*^Diagnosis was made during the pathological examination after death. ^*∗∗*^Diagnosis of the ventricular septal defect was made after birth.

**Table 3:** Management and outcomes of the cases with cardiac diverticulum and pericardial effusion.

	Reference	GA PE	GA Di	Loc.	Size (mm)	Intervention	PE findings	Prenatal progression	Postnatal progression
1	Carles et al. [24]	13	—	Apex LV	—	TOP 14 w	—	—	—

2	Cesko et al. [25]	17	**AP**	Apex RV	3 mm	TOP 22 w	—	—	—

3	McAuliffe et al. [27]	14	14	Apex RV	2-3 mm	TOP	—	—	—

4	Cavallé-Garrido et al. [6]	19	—	RV	3 mm	No	—	Spontaneous resolution at 34 w	Asymptomatic at 22 months

5	Cavallé-Garrido et al. [6]	20	—	LV lateral wall below mitral valve	Large	No	—	Prenatal fetal death at 26 w, trisomy 18	—

6	Prefumo et al. [1]	12	12	Apex LV	1 mm	No	—	Spontaneous resolution, effusion disappeared at 14 weeks; CD was not visible on ultrasound examination from week 21	Asymptomatic at birth effusion or diverticulum not visible; asymptomatic at 17 months' follow-up

7	McAuliffe et al. [27]	13	13	Apex RV	5 × 5 mm	No	—	Spontaneous resolution; CD did not grow Perimembranous IVC	IVC and IAC (postnatal); asymptomatic up to 3 months of age; surgical treatment; asymptomatic at 8 months of age

8	Pradhan et al. [28]	20	20	Apex RV	—	No	—	Spontaneous resolution CD did not grow	Surgical treatment at birth

9	McAuliffe et al. [27]	21	24	RV lateral wall	7 × 10 mm	No	—	Fetal death on week 29	—

10	Perlitz et al. [30]	19	19	Apex LV	—	No	—	No control performed	Full-term live birth; asymptomatic at 10 months of age; heart murmur; no treatment

11	Cavallé-Garrido et al. [6]	19	—	Apex RV	—	PC 20 w	—	No PE relapse, CD did not grow	Full-term live birth; asymptomatic at 12 months of age

12	Carles et al. [24]	19	19	Apex RV	3 mm	PC 20 w	7 cm^3^ yellow fluid, 20 gr/L proteins (transudate), acellular	No PE relapse, CD did not grow	Full-term live birth; asymptomatic at 16 months of age; no treatment

13	Cesko et al. [25]	22	AP	Pared lateral LV	10 × 5 mm	PC 25 w	25 mL old blood fluid	Intrauterine fetal death at 26 weeks (CD rupture)	—

14	Brachlow et al. [23]	13	13	Apex RV	4 × 6 mm	PC 16 w	3 mL serohematic fluid, 18 gr/L proteins (transudate), lymphocytes, and mesothelial cells	No PE relapse or enlarging; CD was not visible on week 37	Full-term live birth; asymptomatic at 10 months of age; no treatment

15	Brachlow et al. [23]	13	13	Apex RV	4 × 3 mm	PC 14 w	0.8 mL serohematic fluid, 15 gr/L proteins (transudate)	No PE relapse; CD did not grow	Full-term live birth; asymptomatic at 8 months of age; no treatment

16	Prefumo et al. [1]	14	14	Apex RV	5 × 5 mm	PC 16 w	5 mL clear fluid	No PE relapse; CD did not grow; mild cardiomegaly	Full-term live birth; asymptomatic at 22 months of age; no treatment

17	Gradiner et al. [19]	14	14	Apex RV	2-3 mm	PC 14 w	2 mL yellow fluid	No PE relapse; CD did not grow	Full-term live birth; asymptomatic; no treatment

18	Carrard et al. [32]	13	15	Apex RV	2.6 × 2.9	PC 17 w	4 mL clear fluid, 21 g/L proteins (transudate)	No PE relapse; diverticulum was not visible from week 26 on	Full-term live birth; asymptomatic at 11 months of age; no treatment

19	Williams et al. [3]	21	21	Apex RV	5 × 4.5	PC 24 w	Yellow fluid 10 mL, 15.4 g/L proteins (transudate), lymphocytes	Complete resolution one week after PC CD did not grow	Full-term live birth; asymptomatic at one year of age; no treatment

20	Barberato et al. [29]	16	16	—	—	PC 20 w	Blood-stained fluid	Moderate growth of PE size as compared with postpuncture effusion; expectant approach Intrauterine fetal death on week 37	—

21	Abi-Nader et al. [2]	12	22	Apex RV		PC 18 w	—	Relapse one week later and subsequent spontaneous resolution on weeks 32-33	—

22	Our case	12	14	Apex RV	2 mm	PC 17 w	Clear yellow fluid, acellular, transudate	No PE relapse; CD did not grow; moderate cardiomegaly	Full-term live birth; asymptomatic at birth; treatment with ASA; asymptomatic at 4 years of age

GA PE: gestational age at pericardial effusion; GA di: gestational age at diverticulum diagnosis; RV: right ventriculum, LV: left ventriculum; w: weeks of pregnancy; PC: pericardiocentesis; CD: cardiac diverticulum; IVC: interventricular communication; PE: pericardial effusion.
