# Corrigendum to “Prenatal Diagnosis of Cardiac Diverticulum with Pericardial Effusion in the First Trimester of Pregnancy with Resolution after Early Pericardiocentesis”

**DOI:** 10.1155/2017/6207658

**Published:** 2017-03-05

**Authors:** Raquel Garcia Rodriguez, Azahara Rodriguez Guedes, Raquel Garcia Delgado, Lourdes Roldan Gutierrez, Margarita Medina Castellano, Jose Angel Garcia Hernandez

**Affiliations:** Prenatal Diagnosis and Fetal Therapy Unit, Department of Obstetrics and Gynecology, University Maternity Hospital of Canaries, Las Palmas, 35004 Gran Canaria, Spain

 In the article titled “Prenatal Diagnosis of Cardiac Diverticulum with Pericardial Effusion in the First Trimester of Pregnancy with Resolution after Early Pericardiocentesis” [1], there were errors in Tables 2 and 3 and in the text citations of some references in the Discussion.

The errors in the in-text citations of references in the Discussion should be corrected as follows:

The original text: Ultrasonographic findings associated with diverticula include pericardial effusion, cardiomegaly, septal defects and arrhythmia with fetal death before delivery, and hydrops [6,13,14].

The corrected text: Ultrasonographic findings associated with diverticula include pericardial effusion, cardiomegaly, septal defects and arrhythmia with fetal death before delivery, and hydrops [6, 13, 28, 32].

The original text: Thus, the observation of pericardial effusion makes it necessary to examine the cardiac function [1, 6, 15].

The corrected text: Thus, the observation of pericardial effusion makes it necessary to examine the cardiac function [1, 6, 16].

The original text: Five of them showed spontaneous resolution (71%) and 2 resulted in intrauterine death (29%): one of them, which occurred on week 26, was associated with trisomy 18 and the other, which occurred on week 29, was associated with treated twin-to-twin transfusion syndrome and death of one of the twins after treatment [6, 16].

The corrected text: Five of them showed spontaneous resolution (71%) and 2 resulted in intrauterine death (29%): one of them, which occurred in week 26, was associated with trisomy 18 and the other, which occurred in week 29, was associated with treated twin-to-twin transfusion syndrome and death of one of the twins after treatment [6, 17].

The original text: The prognosis of this entity is generally good, although the outcome largely depends on the size and location of associated anomalies. Cases of rupture, both pre- and postnatal, arrhythmia, fetal death, heart failure, and coronary insufficiency have been described [9, 16, 18,21, 23]. In these patients, serial control examinations are necessary to detect possible complications. In general, postnatal progression is good and surgery is not necessary in asymptomatic cases [19].

The corrected text: The prognosis of this entity is generally good, although the outcome largely depends on the size and location of associated anomalies. Cases of rupture, both pre- and postnatal, arrhythmia, fetal death, heart failure, and coronary insufficiency have been described [9, 16, 17,28, 29]. In these patients, serial control examinations are necessary to detect possible complications. In general, postnatal progression is good and surgery is not necessary in asymptomatic cases [18].

Errors in Table 2 should be corrected as follows.

  Row 25: Williams et al. (2009) [3] should be Abi-Nader et al. (2009) [2].
  Rows 29, 30, and 31: Abi-Nader et al. (2009) [2] should be Williams et al. (2009) [3].
  Row 32: Williams et al. (2009) [3] should be Paoletti et al. (2012) [20].
  Row 33: Paoletti and Robertson (2012) [20] should be Nam et al. (2010) [21].
  Row 34: Nam et al. (2010) [21] should be Olorón et al. (2011) [22].

Errors in Table 3 should be corrected as follows.

  Rows 4 and 11: Cavallé-Garrido et al.: the reference in the bibliography is [6].
  Row 7: McAuliffe et al. [27] should be Del Río et al. [18].
  Row 8: Pradhan et al. [28] should be Davidson et al. [15].
  Row 9: McAuliffe et al. [27] should be Koshiishi et al. [17].
  Row 10: Perlitz et al. [30] should be Menahem [31].
  Row 12: Carles et al. [24] should be Johnson et al. [16].
  Row 13: Cesko et al. [25] should be Bernasconi et al. [26].
  Rows 14 and 15: Brachlow et al. [23] should be McAuliffe et al. [27].
  Row 19: Williams et al. [3] should be Abi-Nader et al. [2].
  Row 21: Abi-Nader et al. [2] should be Williams et al. [3].

The corrected tables are shown in Tables 2 and 3.

## Figures and Tables

**Table 2:** Description of the cases of cardiac diverticulum reported in the literature.

	Author	GA di	Size	Sex	Location	Karyotype	Associated anomalies	Intervention	Prenatal progression	Neonatal	Follow-up
1	Kitchiner et al. (1990) [13]	33	—	Female	Apex VI	—	Cardiomegaly	No	Stable	Vaginal delivery 40 w; cardiomegaly, tachypnea, heart murmur, muscular IVC, mild mitral regurgitation	Asymptomatic at 3.5 months of life

2	Hornberger et al. (1994) [9]	31	—	—	Lateral wall below tricuspid valve (RV)	—	—	No	—	—	—

3	Carles et al. (1995) [24]	13	—	Male	Apex LV	—	Pericardial effusion	TOP 14 w	—	—	—

4	Cesko et al. (1998) [25]	17	—	Male	Apex RV	46XY	Pericardial effusion	TOP 22 w	Stable	—	—

5	Cavallé-Garrido et al. (1997) [6]	20	Large	Female	Lateral wall below mitral valve (LV)	Trisomy 18	Ventricular septal defect, hydrops	No	Fetal death 26 w	—	—

6	Cavallé-Garrido et al. (1997) [6]	19	Small	Female	Apex RV	—	No	No	Stable; spontaneous resolution at 34 w	Asymptomatic	Asymptomatic at 22 months of life

7	Cavallé-Garrido et al. (1997) [6]	19	Small	—	Apex RV	—	Pericardial effusion	PC 20 w	Stable	Asymptomatic	Asymptomatic at 12 months of life

8	Cavallé-Garrido et al. (1997) [6]	36	Small	Male	Lateral wall below tricuspid valve (RV)	—	Pericardial effusion	—	—	—	Asymptomatic at 18 months of life

9	Johnson et al. (1996) [16]	19	3 mm	Female	Apex RV	46XX	Pericardial effusion	PC 20 w	No relapse after PC, no growth	Eutocic delivery 41 w; weight 3700 grams Asymptomatic	Asymptomatic at 16 months of life

10	Brachlow et al. (2002) [23]	32	—	—	Apex LV	—	Cardiomegaly	No	Stable	—	Asymptomatic at 6 months of life

11	Bernasconi et al. (2004) [26]	22	10 × 5 mm	Male	LV lateral wall below mitral valve^*∗*^	46XY	Pericardial effusion	PC 22 w	—	Fetal death 26 w, probably due to diverticulum rupture	—

12	McAuliffe et al. (2005) [27]	13	4 × 6 mm	Male	Apex RV	46XY	First trimester NT 4.2 mm Pericardial effusion	PC 16 w	Resolution of the effusion; CD stable	Eutocic delivery 38 w; weight of 3070 grams Asymptomatic	Asymptomatic at 10 months of life

13	McAuliffe et al. (2005) [27]	13	4 × 3 mm	Male	Apex RV	46XY	First trimester NT 2 mm Pericardial effusion	PC 14 w	Resolution of the effusion; CD stable	Eutocic delivery 38 w; weight 3150 grams Asymptomatic	Asymptomatic at 8 months of life

14	Prefumo et al. (2005) [1]	14	5 × 5	Male	Apex RV	46XY	First trimester NT 3.7 mm Pericardial effusion, ascites, skin edema	PC 16 w	Resolution of the effusion and hydrops; CD stable; mild cardiomegaly	Vaginal full-term eutocic delivery; asymptomatic	Asymptomatic at 22 months of life

15	Prefumo et al. (2005) [1]	12	1 mm	—	Apex RV	—	First trimester NT 1.2 mm Pericardial effusion	No	Spontaneous resolution of PE with 21 w; CD stable	Full-term eutocic delivery, asymptomatic	Asymptomatic at 17 months of life

16	Gardiner et al. (2009) [19]	14	2-3 mm	—	Apex RV	Normal	Pericardial effusion	PC 14 w	Resolution of the effusion and hydrops CD collapsed	Asymptomatic at birth	—

17	Gardiner et al. (2009) [19]	14	2-3 mm	—	Apex RV	Normal	Pericardial effusion	TOP	—	—	—

18	Del Río et al. (2005) [18]	13	5 × 5	Female	Apex RV	46XX	Pericardial effusion, septal defect AV^*∗∗*^	No	Spontaneous resolution at 28 w	Eutocic delivery 40 w; weight 3400 grams, asymptomatic at birth	Correction of septal defect at 3 months of life, resection of diverticulum; Asymptomatic at 8 months of life

19	Wax et al. (2007) [14]	20	6 × 9 mm	Male	Junction base RV-infundibulum	—	No	No	Stable	Full-term eutocic delivery; Weight 3689 grams, asymptomatic; small permeable FO	Asymptomatic at 18 months of life

20	Koshiishi et al. (2007) [17]	24	7 × 10 mm	—	Lateral wall below tricuspid valve (RV)	—	Mild pericardial effusion; MC pregnancy with laser intervention for TTTS at week 20 where donor fetus died	No	Stable	Prenatal fetal death at 29 w	—

21	Pradhan et al. (2007) [28]	28	—	—	Apex LV	—	Fetal arrhythmia Hydrops fetalis	Medical treatment (digoxin)	—	Vaginal delivery 40 w	Asymptomatic at 12 months of life

22	Barberato et al. (2009) [29]	16	5 × 5,7 mm	—	Apex LV	—	Mild pericardial effusion	PC 20 w	Discrete enlargement of PE with normal heart function	Prenatal fetal death 37 w	—

23	Barberato et al. (2009) [29]	30	12 × 13 mm	—	Mitral subvalvular	—	LV dilatation and reduced systolic function	No	Stable	—	Asymptomatic at 6 months of life

24	Davidson et al. (2009) [15]	20	—	—	Apex RV	—	Pericardial effusion	No	Spontaneous resolution	—	Surgical treatment

25	Abi-Nader et al. (2009) [2]	21	5 × 5,5 mm	Male	RV	—	Pericardial effusion	PC 24 w	Mild tricuspid regurgitation at 31 CD stable	Full-term delivery	Asymptomatic at a year of life

26	Perlitz et al. (2009) [30]	22	7 × 4 mm	Male	RV lateral wall	—	No	No	Stable, CD growth up to 9 × 9 mm	Eutocic delivery week 40; weight 4010 grams Asymptomatic at birth	Asymptomatic at a year of life

27	Menahem (2010) [31]	19	—	—	Apex LV	—	Pericardial effusion	—	No controls performed	Full-term live birth	Asymptomatic at 10 months of life

28	Carrard et al. (2010) [32]	13	2,6 × 2,9 mm	Male	RV lateral wall	46XY	First trimester NT 2.2 mm Pericardial effusion	PC 17 w	Resolution after PC; CD collapsed at 26 w	Eutocic delivery 40 w, 2780 grams	Asymptomatic at 11 months of life

29	Williams et al. (2009) [3]	22	3-4 mm	Male	RV	46XY	Pericardial effusion	PC 18 w	Reaccumulation after treatment and resolution at 32-33 w	PROM 34 w; intubation due to prematurity; caesarean section; weight 2460 gr; 2 muscle IVCs	Asymptomatic at 14 months of life

30	Williams et al. (2009) [3]	21	11 × 15 mm	Male	RV lateral wall below tricuspid valve	—	Isolated	—	—	Eutocic delivery; weight 2780 gr; asymptomatic at birth	Asymptomatic at 16 months of life

31	Williams et al. (2009) [3]	25	26 × 16 mm (37 s)	Male	RV	—	Arrhythmia and reduced systolic function	Induced delivery	—	Caesarean section 38 + 5 w; weight 3270 grams; mild reduction of systolic function and premature ventricular contractions at birth	Asymptomatic at 3 years of life, on prophylactic treatment with acetyl salicylic acid

32	Paoletti and Robertson (2012) [20]	17	—	—	Apex LV	Normal	Mesocardia, per-membranous IVC	No	Stable	Full-term live birth	Asymptomatic at 2 years of life

33	Nam et al. (2012) [21]	21	1,6 × 0,4 mm	—	Apex LV	Normal	Defect on thoracoabdominal midline	TOP	—	—	—

34	Olorón et al. (2011) [22]	31	12 mm (postnatal)	—	RV lateral wall below tricuspid valve	—	—	No	Ventricular septal defect	Full-term live birth; asymptomatic at birth; symptoms at 45 days of life: closure of septal defect at 3 months of life	Asymptomatic at 10 months of life

35	Our case	14	2 mm	Male	Apex RV	46XY	Pericardial effusion	PC 17 w	PE resolution after treatment; CD Stable; moderate cardiomegaly; normal heart function	Full-term live birth; spontaneous eutocic delivery 40 + 1 w; weight 3150 grams	Asymptomatic at 4 years of life

GA di: gestational age at diagnosis; RV: right ventriculum; LV: left ventriculum; w: weeks of pregnancy; TOP: termination of pregnancy; PC: pericardiocentesis; CD: cardiac diverticulum; IVC: interventricular communication; PE: pericardial effusion; PROM: premature rupture of membranes; NT: nuchal translucency.

^*∗*^Diagnosis was made during the pathological examination after death. ^*∗∗*^Diagnosis of the ventricular septal defect was made after birth.

**Table 3:** Management and outcomes of the cases with cardiac diverticulum and pericardial effusion.

	Reference	GA PE	GA di	Loc.	Size (mm)	Intervention	PE findings	Prenatal progression	Postnatal progression
1	Carles et al. [24]	13	—	Apex LV	—	TOP 14 w	—	—	—

2	Cesko et al. [25]	17	**AP**	Apex RV	3 mm	TOP 22 w	—	—	—

3	Gardiner et al. [27]	14	14	Apex RV	2-3 mm	TOP	—	—	—

4	Cavallé-Garrido et al. [6]	19	—	RV	3 mm	No	—	Spontaneous resolution at 34 w	Asymptomatic at 22 months

5	Cavallé-Garrido et al. [6]	20	—	LV lateral wall below mitral valve	large	No	—	Prenatal fetal death at 26 w, trisomy 18	—

6	Prefumo et al. [1]	12	12	Apex LV	1 mm	No	—	Spontaneous resolution, effusion disappeared at 14 weeks; CD was not visible on ultrasound examination from week 21	Asymptomatic at birth; effusion or diverticulum not visible Asymptomatic at 17-month follow-up

7	Del Río et al. [18]	13	13	Apex RV	5 × 5 mm	No	—	Spontaneous resolution; CD did not grow Perimembranous IVC	IVC and IAC (postnatal) Asymptomatic up to 3 months of age; surgical treatment Asymptomatic at 8 months of age

8	Davidson et al. [15]	20	20	Apex RV	—	No	—	Spontaneous resolution; CD did not grow	Surgical treatment at birth

9	Koshiishi et al. [17]	21	24	RV lateral wall	7 × 10 mm	No	—	Fetal death on week 29	—

10	Menahem [31]	19	19	Apex LV	—	No		No control performed	Full-term live birth; asymptomatic at 10 months of age; heart murmur; no treatment

11	Cavallé-Garrido et al. [6]	19	—	Apex RV	—	PC 20 w	—	No PE relapse, CD did not grow	Full-term live birth; asymptomatic at 12 months of age

12	Johnson et al. [16]	19	19	Apex RV	3 mm	PC 20 w	7 cm^3^ yellow fluid, 20 gr/L proteins (transudate), acellular	No PE relapse, CD did not grow	Full-term live birth; asymptomatic at 16 months of age; no treatment

13	Bernasconi et al. [26]	22	AP	Pared lateral LV	10 × 5 mm	PC 25 w	25 mL old blood fluid	Intrauterine fetal death at 26 weeks (CD rupture)	—

14	McAuliffe et al. [27]	13	13	Apex RV	4 × 6 mm	PC 16 w	3 mL serohematic fluid, 18 gr/L proteins (transudate), lymphocytes and mesothelial cells	No PE relapse or enlarging; CD was not visible on week 37	Full-term live birth; asymptomatic at 10 months of age; no treatment

15	McAuliffe et al. [27]	13	13	Apex RV	4 × 3 mm	PC 14 w	0.8 mL serohematic fluid, 15 gr/L proteins (transudate)	No PE relapse; CD did not grow	Full-term live birth; asymptomatic at 8 months of age; no treatment

16	Prefumo et al. [1]	14	14	Apex RV	5 × 5 mm	PC 16 w	5 mL clear fluid	No PE relapse; CD did not grow; mild cardiomegaly	Full-term live birth; asymptomatic at 22 months of age; no treatment

17	Gardiner et al. [19]	14	14	Apex RV	2-3 mm	PC 14 w	2 mL yellow fluid	No PE relapse; CD did not grow	Full-term live birth; asymptomatic; no treatment

18	Carrard et al. [32]	13	15	Apex RV	2.6 × 2.9	PC 17 w	4 mL clear fluid, 21 g/L proteins (transudate)	No PE relapse; diverticulum was not visible from week 26 on	Full-term live birth; asymptomatic at 11 months of age; no treatment

19	Abi-Nader et al. [2]	21	21	Apex RV	5 × 4.5	PC 24 w	Yellow fluid 10 mL, 15.4 g/L proteins (transudate), lymphocytes	Complete resolution one week after PC; CD did not grow	Full-term live birth; asymptomatic at one year of age; no treatment

20	Barberato et al. [29]	16	16	—	—	PC 20 w	Blood-stained fluid	Moderate growth of PE size as compared with postpuncture effusion; expectant approach; intrauterine fetal death on week 37	—

21	Williams et al. [3]	12	22	Apex RV	—	PC 18 w	—	Relapse one week later and subsequent spontaneous resolution on week 32-33	—

22	Our case	12	14	Apex RV	2 mm	PC 17 w	Clear yellow fluid, acellular, transudate	No PE relapse; CD did not grow	Full-term live birth; asymptomatic at birth; treatment with ASA; asymptomatic at 4 years of age

GA PE: gestational age at pericardial effusion; GA di: gestational age and diverticulum diagnosis; RV: right ventriculum, LV: left ventriculum; w: weeks of pregnancy; PC: pericardiocentesis; CD: cardiac diverticulum; IVC: interventricular communication; PE: pericardial effusion.
